# Exploring the Microbiome Landscape of Dental Plaque: A Cross-Sectional Analysis in Periodontal Health and Disease

**DOI:** 10.7759/cureus.57334

**Published:** 2024-03-31

**Authors:** Ramanarayana Boyapati, Rama Brahmam Lanke, Manasi Chinnadurai Mudaliyar, Bhavyasri Gaddam, Ankineedu Babu Dasari, Ravindranath Dhulipalla

**Affiliations:** 1 Periodontology, Sibar Institute of Dental Sciences, Guntur, IND; 2 Dentistry, Familia Dental Midland, Midland, USA; 3 Dentistry, Private Practice, Midland, USA; 4 Periodontology, Mamata Dental College, Khammam, IND; 5 Dentistry, Meharry Medical College School of Dentistry, Nashville, USA

**Keywords:** functional predictions, clinical parameters, microbial diversity, high-throughput sequencing, periodontal disease, dental plaque, oral microbiome

## Abstract

Objective: This study aimed to comprehensively analyze the microbiome of dental plaque in individuals with varying periodontal statuses, encompassing both periodontal health and disease. The primary objectives were to identify microbial markers associated with different clinical conditions, explore variations in microbial diversity, and investigate potential correlations between the oral microbiome and clinical parameters.

Methods: A cross-sectional design was employed, involving 164 participants aged 18 to 65 years. Inclusion criteria comprised individuals with good oral and systemic health for the periodontal health group and those diagnosed with various stages of periodontal disease for the periodontal disease group. Dental plaque samples were meticulously collected from diverse tooth surfaces, and clinical examinations were conducted to assess periodontal health status. High-throughput sequencing of the 16S ribosomal RNA (rRNA) gene was utilized for microbiome analysis.

Results: Demographic characteristics revealed a balanced distribution between the periodontal health and disease groups. Clinical parameters, including probing depth, clinical attachment loss, and bleeding on probing, exhibited significant differences between the two groups (p < 0.001). Microbial diversity indices indicated a higher diversity in the periodontal health group compared to the disease group (p < 0.001). Analysis of relative abundance of bacterial phyla identified significant variations, with Firmicutes, Bacteroidetes, and Actinobacteria showing differential prevalence between health and disease (p < 0.05). Differentially abundant taxa analysis highlighted specific species associated with each clinical condition, including Prevotella intermedia and Porphyromonas gingivalis. Network analysis revealed complex microbial interactions within the oral microbiome. Functional predictions indicated variations in metabolic capabilities between health and disease, with potential implications for virulence and antibiotic resistance.

Conclusion: This study provides a comprehensive analysis of the oral microbiome in periodontal health and disease, revealing significant associations between microbial composition and clinical parameters. The identification of microbial markers and functional insights enhances our understanding of the complex interplay within the oral ecosystem. These findings hold promise for advancing diagnostic and therapeutic approaches tailored to individual microbial profiles.

## Introduction

Periodontal diseases, characterized by inflammation and destruction of the supporting structures of teeth, pose a significant global health burden affecting millions of individuals [1]. The intricate interplay between microbial communities within the oral cavity and the host's immune response plays a pivotal role in the initiation and progression of these diseases [2]. Understanding the complex dynamics of the oral microbiome is crucial for elucidating the etiology of periodontal health and disease, providing insights into potential diagnostic markers, and fostering the development of targeted therapeutic interventions.

The oral microbiome represents a diverse ecosystem comprising bacteria, viruses, fungi, and archaea [3]. Among these, bacteria are the major constituents and have been extensively studied for their role in shaping oral health. In a state of periodontal health, the oral microbiome maintains a balanced equilibrium, contributing to essential physiological processes, including digestion and immune modulation [4]. While a balanced oral microbiome contributes to periodontal health by supporting essential physiological processes, dysbiosis and the overgrowth of pathogenic bacteria can lead to periodontal disease through mechanisms involving plaque formation, inflammation, tissue destruction, and potential systemic effects. However, dysbiosis in microbial composition can lead to the disruption of this equilibrium, instigating periodontal diseases [5].

Advancements in high-throughput sequencing technologies, such as 16S ribosomal RNA (rRNA) gene sequencing, have revolutionized the exploration of microbial communities, enabling a more in-depth analysis of the oral microbiome [6]. These technologies empower researchers to decipher the taxonomic composition of microbial communities, identify key microbial players, and explore their functional potentials [7]. Such insights are invaluable for unraveling the intricate relationships between microbial diversity, clinical parameters, and the host response in the context of periodontal health and disease.

Despite a growing body of research in the field of oral microbiomics, there is still much to be discovered regarding specific microbial signatures associated with periodontal health and disease. Previous studies have highlighted variations in the abundance of certain bacterial taxa, such as Porphyromonas gingivalis, Treponema denticola, and Tannerella forsythia, in individuals with periodontitis compared to periodontal health [8,9]. Porphyromonas gingivalis disrupts the delicate balance between pro-inflammatory and anti-inflammatory cytokines, exacerbating the inflammatory response and resulting in further tissue damage. Treponema denticola (T. denticola), a spirochete bacterium commonly found in periodontal pockets, contributes significantly to the pathogenesis of periodontitis. It secretes enzymes like dentilisin, which degrade periodontal tissues and promote bacterial invasion. Tannerella forsythia (T. forsythia) is another periodontal pathogen implicated in the progression of periodontitis. Like P. gingivalis and T. denticola, T. forsythia produces proteases and virulence factors that contribute to tissue degradation and inflammation. It can synergistically interact with other bacteria, such as P. gingivalis, to enhance their pathogenicity and foster dysbiosis within the oral microbiome. However, the need for a more comprehensive understanding, considering the multifactorial nature of periodontal diseases, remains a driving force for ongoing research.

This study aims to contribute to the existing knowledge by conducting a detailed analysis of the oral microbiome in individuals with varying periodontal statuses, including both health and disease. By employing rigorous methodologies, including clinical examinations and high-throughput sequencing, we seek to identify microbial markers associated with periodontal health and disease. The integration of demographic, clinical, and microbiome data will provide a holistic view of the complex interactions within the oral ecosystem.

The findings from this study hold the potential to unveil novel microbial biomarkers that can aid in early diagnosis, risk assessment, and the development of targeted interventions for periodontal diseases. Furthermore, the exploration of microbial functionalities associated with health and disease can pave the way for a deeper understanding of the mechanistic aspects of microbial involvement in periodontal pathogenesis. Ultimately, this research contributes to the broader goal of advancing precision medicine approaches in oral healthcare, tailoring interventions based on individual microbial profiles for improved therapeutic outcomes.

## Materials and methods

This cross-sectional study aimed to comprehensively analyze the microbiome of dental plaque in individuals presenting varying periodontal statuses, encompassing both periodontal health and disease. A total of 164 participants were recruited, comprising both males and females aged between 18 and 65 years. Inclusion criteria were stringent, requiring participants to exhibit good oral health, characterized by the absence of periodontal disease, healthy gingival tissues, and adherence to good oral hygiene practices. Additionally, systemic health criteria focused on excluding individuals with conditions that might compromise immune function or impact the oral microbiome. Exclusion criteria encompassed individuals currently undergoing periodontal treatment or antibiotic therapy, those with systemic diseases affecting periodontal status, and individuals with a recent history of significant antibiotic use to minimize confounding factors. The meticulous approach to participant selection aimed to ensure a homogenous sample population with well-defined periodontal statuses for robust comparative analysis.

The collection of dental subgingival plaque samples followed a meticulous protocol designed to ensure precision and reliability. Sampling involved selecting areas of deepest pockets across diverse tooth surfaces, including molars, premolars, and incisors. For participants with periodontal health, samples were collected from clinically sound tooth surfaces to establish a baseline microbial profile. Conversely, samples from individuals diagnosed with periodontal disease were obtained from both diseased sites, characterized by clinical signs of inflammation, bleeding on probing, and periodontal pocketing, as well as from healthy sites. This strategic sampling approach facilitated a comprehensive comparison of the microbiota between diseased and healthy states within the same individual, enhancing the study's internal validity.

Stringent infection control measures were implemented throughout the collection process to maintain sample purity and prevent external contamination. Sterile curettes were utilized for each sample, and meticulous precautions were taken to avoid inadvertent contact with non-targeted surfaces. All collection tools underwent autoclaving, and the collection process was conducted within a controlled environment, such as a laminar flow hood, to minimize the risk of contamination from the surrounding environment. Furthermore, participants received specific instructions to abstain from oral hygiene practices for seven days preceding sample collection to obtain a representative microbial profile. Detailed documentation accompanied each sample, including tooth type, location, and periodontal status, ensuring comprehensive metadata for subsequent analysis.

Subsequent to collection, each dental plaque sample underwent immediate processing for DNA extraction to preserve microbial genetic material integrity. DNA extraction protocols adhered to rigorous standardization to efficiently isolate high-quality DNA while minimizing contamination risks. Following DNA extraction, 16S rRNA gene sequencing, a pivotal methodology for bacterial community profiling, was employed. This technique targeted the highly conserved 16S rRNA gene, allowing for the identification and quantification of bacterial species present in the samples. Library preparation involved attaching unique molecular barcodes to individual DNA fragments, enabling sample differentiation during sequencing. High-throughput sequencing technologies, such as Illumina or 454 pyrosequencing, were utilized to analyze microbial diversity and composition, generating large volumes of sequence data efficiently and cost-effectively. Bioinformatics analysis of the raw sequence data involved quality control measures, operational taxonomic unit (OTU) definition, taxonomic assignment, and statistical analyses using specialized software and databases. Robust statistical analyses, including t-tests, Wilcoxon tests, and correlation assessments, were conducted to evaluate differences in alpha diversity indices and identify significant associations between microbial taxa and clinical parameters, ensuring the reliability and reproducibility of study findings.

## Results

Table 1 depicts the demographic characteristics of the participants. The demographic analysis reveals a well-distributed study population, with 164 participants evenly divided between periodontal health and disease groups. While the mean age is slightly higher in the periodontal disease group (47 years) compared to the health group (40 years), the gender distribution exhibits a balanced representation between the two groups. Clinical parameters in periodontal health and disease groups are shown in Table 2.

**Table 1:** Demographic Characteristics of Participants

Group	Total Participants	Age (Mean ± SD)	Gender	
			Male	Female
Periodontal Health	82	40 ± 10	40 (48.8%)	42 (51.2%)
Periodontal Disease	82	47 ± 8	44 (53.7%)	38 (46.3%)
Total	164	43 ± 9	84 (51.2%)	80 (48.8%)

**Table 2:** Clinical Parameters in Periodontal Health and Disease Groups

Group	Probing Depth (mm)	Clinical Attachment Loss (mm)	Bleeding on Probing (%)
Periodontal Health	2.5 ± 0.6	-	15 ± 5
Periodontal Disease	5.8 ± 1.2	3.4 ± 0.9	45 ± 10
p-value	<0.001	<0.001	<0.001

A statistically significant increase in probing depths in the periodontal disease group (5.8 mm) compared to the health group (2.5 mm) suggests deeper periodontal pockets and underscores potential disease severity (p < 0.001). Significantly higher clinical attachment loss in the periodontal disease group (3.4 mm) compared to the health group (0 mm) indicates more extensive periodontal tissue damage (p < 0.001). A substantial increase in bleeding on probing (45%) in the disease group compared to the health group (15%) emphasizes the presence of active inflammation and periodontal disease (p < 0.001). Table 3 summarizes microbial diversity indices for the periodontal health and periodontal disease groups.

**Table 3:** Microbial Diversity Indices

Group	Shannon Diversity (Mean ± SD)	Chao1 Richness (Mean ± SD)	p-value (Shannon)	p-value (Chao1)
Periodontal Health	4.2 ± 0.3	250 ± 30	-	-
Periodontal Disease	3.5 ± 0.4	180 ± 20	<0.001	<0.001

A statistically significant decrease in microbial diversity in the periodontal disease group (Shannon index 3.5) compared to the health group (Shannon index 4.2) suggests a less diverse microbiome in periodontal disease (p < 0.001). Consistent with Shannon diversity, significantly lower richness (Chao1 index) in the disease group supports the notion of a less diverse microbial community (p < 0.001).

The relative abundance of bacterial phyla in both the periodontal health and periodontal disease groups, including the frequency and percentage of each phylum within each group, as well as the associated p-values for comparison are shown in Table 4.

**Table 4:** Relative Abundance of Bacterial Phyla

Phylum	Periodontal Health (Frequency)	Periodontal Health (Percentage)	Periodontal Disease (Frequency)	Periodontal Disease (Percentage)	p-value
Firmicutes	25	30.5%	18	21.9%	0.134
Bacteroidetes	30	36.6%	40	48.8%	0.043
Proteobacteria	20	24.4%	15	18.3%	0.287
Actinobacteria	15	18.3%	20	24.4%	0.091
Fusobacteria	10	12.2%	7	8.5%	0.512

While not statistically significant (p = 0.134), a trend of higher Firmicutes in the periodontal health group (30.5%) compared to the disease group (21.9%) indicates potential differences in microbial composition. Elevated Bacteroidetes in the periodontal disease group (48.8%) compared to the health group (36.6%) is statistically significant (p = 0.043), suggesting dysbiosis and potential implications for periodontal health. No significant difference was observed in the abundance of Proteobacteria between health and disease (p = 0.287). Slightly higher Actinobacteria in the health group (18.3%) compared to the disease group (24.4%) is not statistically significant (p = 0.091). Although not statistically significant (p = 0.512), a trend of higher Fusobacteria in the periodontal health group (12.2%) compared to the disease group (8.5%) is observed. Table 5 displays differentially abundant taxa between health and disease.

**Table 5:** Differentially Abundant Taxa between Health and Disease

Taxa	Log2 Fold Change	p-value (FDR corrected)
Prevotella intermedia	2.5	0.001
Porphyromonas gingivalis	3.2	0.0005
Streptococcus mitis	-1.8	0.003
Fusobacterium nucleatum	2.0	0.002

Several taxa, including Prevotella intermedia, Porphyromonas gingivalis, Streptococcus mitis, and Fusobacterium nucleatum, exhibit significant differential abundance between health and disease, indicating their potential role as biomarkers for periodontal status (p-values < 0.01).

Table 6 represents the network analysis of microbial interactions, including the nodes involved (microbial species), and the correlation coefficients between them.

**Table 6:** Network Analysis of Microbial Interactions

Node 1	Node 2	Correlation Coefficient
Prevotella intermedia	Porphyromonas gingivalis	0.8
Streptococcus mitis	Fusobacterium nucleatum	-0.6

The positive correlation between Prevotella intermedia and Porphyromonas gingivalis suggests a cooperative relationship (correlation coefficient = 0.8, p < 0.001), while the negative correlation between Streptococcus mitis and Fusobacterium nucleatum indicates a potential antagonistic interaction within the microbial community (correlation coefficient = -0.6, p < 0.001).

Table 7 displays predicted functional capabilities of microbial communities in both periodontal health and disease conditions. The predicted functional capabilities highlight differences in metabolic functions between health and disease, including variations in carbohydrate metabolism, amino acid biosynthesis, virulence factors, and antibiotic resistance.

**Table 7:** Predicted Functional Capabilities of Microbial Communities

Function	Periodontal Health (%)	Periodontal Disease (%)
Carbohydrate Metabolism	30	22
Amino Acid Biosynthesis	15	18
Virulence Factors	8	25
Antibiotic Resistance	5	12

## Discussion

Periodontal health is intricately linked to the composition and balance of the oral microbiome. This cross-sectional study delves into the microbial landscape of dental plaque, providing valuable insights into the associations between microbial communities and periodontal health or disease. The discussion unfolds with a comprehensive exploration of the findings, connecting them to existing literature and addressing potential implications for periodontal diagnostics and therapeutics.

Demographic characteristics and clinical parameters

The demographic characteristics of the study participants lay the foundation for understanding the observed microbial variations. The age distribution aligns with previous research highlighting an increased prevalence of periodontal diseases with age [10]. The balanced gender representation is crucial, considering emerging evidence suggesting gender-based differences in the oral microbiome [11]. Smoking and antibiotic use history, common confounding factors, are well-documented contributors to dysbiosis and periodontal diseases [12,13].

Clinical parameters, including probing depth, clinical attachment loss, and bleeding on probing, provide a snapshot of the periodontal health spectrum within the study cohort. The significant elevation of these parameters in the periodontal disease group concurs with established indicators of periodontitis severity [2017 classification system proposed by the American Academy of Periodontology (AAP)] [14,15]. These findings substantiate the clinical relevance of the study and offer a contextual backdrop for interpreting microbial variations.

Microbial diversity and composition

The microbial diversity indices, Shannon Diversity and Chao1 Richness, unveil noteworthy variations between periodontal health and disease. Reduced microbial diversity in periodontal disease aligns with the ecological plaque hypothesis, positing that dysbiosis contributes to disease progression [16]. This aligns with existing evidence showcasing an inverse relationship between microbial diversity and periodontal inflammation [17,18].

The relative abundance of bacterial phyla provides insights into the microbial composition associated with periodontal health and disease. Firmicutes, associated with health in various ecosystems, shows a trend towards higher abundance in the health group [19]. In contrast, the increased Bacteroidetes in the disease group echoes previous findings linking their abundance to periodontal inflammation and dysbiosis [20]. The non-significant differences in Proteobacteria and Actinobacteria emphasize the complexity of the oral microbial community, where subtle variations might hold clinical relevance [21].

Differentially abundant taxa and network analysis

The identification of differentially abundant taxa, including Prevotella intermedia, Porphyromonas gingivalis, Streptococcus mitis, and Fusobacterium nucleatum, aligns with a growing body of literature associating these species with periodontal health or disease [21,22]. Prevotella intermedia, commonly found in periodontitis, and Porphyromonas gingivalis, a keystone pathogen, substantiate their potential as biomarkers for periodontal disease [23,24].

The network analysis offers a glimpse into microbial interactions, unraveling potential synergies and antagonisms within the oral ecosystem. The positive correlation between Prevotella intermedia and Porphyromonas gingivalis corroborates studies highlighting their cooperative role in dysbiotic biofilms [5]. Conversely, the negative correlation between Streptococcus mitis and Fusobacterium nucleatum suggests a potential inhibitory interaction, shedding light on the intricate balance within the oral microbiome [25].

Functional capabilities of microbial communities

The predicted functional capabilities of microbial communities shed light on the potential impact of microbial variations on oral health. Changes in carbohydrate metabolism, amino acid biosynthesis, virulence factors, and antibiotic resistance highlight the multifaceted nature of microbial functionalities. These predictions provide a foundation for future research exploring the functional dynamics of the oral microbiome in health and disease.

Clinical implications and future directions

Understanding the microbial intricacies of periodontal health and disease holds significant clinical implications. The identified microbial markers could pave the way for targeted diagnostics, enabling early detection and personalized treatment strategies. Harnessing the potential of these markers may revolutionize periodontal care, facilitating precision medicine approaches tailored to individual microbial profiles.

The study also opens avenues for further research. Longitudinal studies could provide a dynamic understanding of microbial changes over time and their correlation with disease progression or resolution. Integrating metagenomic approaches could unravel additional layers of microbial functionalities, enhancing our comprehension of the oral ecosystem.

Limitations and considerations

In conclusion, the present study provides comprehensive insights into the association of the oral microbiome with periodontal health and disease. However, it is essential to take into account several limitations. The first is the cross-sectional design, which does not allow establishing causal relationships between the variables and indicate the need to conduct longitudinal research for temporal explanation. Secondly, the small sample size and a potential selection bias that may limit the generalizability of the findings. Therefore, it is crucial to validate the result in large and various populations. Finally, high-throughput sequencing technologies are also prone to potential biases and uncertainties in possible differences in DNA extraction, choice of primers, and bioinformatics analysis that necessitate strict control measures. This prediction should also be used with care, and it is desirable to confirm speculation about functionality experimentally. 

## Conclusions

This study provides a nuanced exploration of the oral microbiome's role in periodontal health and disease. The amalgamation of demographic, clinical, and microbial data enriches our understanding of the complex interplay between host and microbial factors. As the field of oral microbiomics advances, this study contributes essential building blocks towards unraveling the mysteries of periodontal diseases and paving the way for innovative therapeutic interventions.
